# Panduratin A from *Boesenbergia rotunda* Effectively Inhibits EGFR/STAT3/Akt Signaling Pathways, Inducing Apoptosis in NSCLC Cells with Wild-Type and T790M Mutations in EGFR

**DOI:** 10.3390/ijms26052350

**Published:** 2025-03-06

**Authors:** Wanna Eiamart, Piyanuch Wonganan, Sarin Tadtong, Weerasak Samee

**Affiliations:** 1 Department of Pharmaceutical Chemistry, Faculty of Pharmacy, Srinakharinwirot University, Nakhon Nayok 26120, Thailand; wanna.eiamart@g.swu.ac.th; 2 Chula Pharmacokinetic Research Center, Faculty of Medicine, Chulalongkorn University, Bangkok 10330, Thailand; 3 Department of Pharmacology, Faculty of Medicine, Chulalongkorn University, Bangkok 10330, Thailand; piyanuch.w@chula.ac.th; 4 Department of Pharmacognosy, Faculty of Pharmacy, Srinakharinwirot University, Nakhon Nayok 26120, Thailand; sarin@g.swu.ac.th

**Keywords:** panduratin A, non-small cell lung cancer, apoptosis, EGFR, STAT3, Akt, molecular docking, ADMET prediction

## Abstract

Non-small cell lung cancer (NSCLC) is a challenging disease, with the epidermal growth factor receptor (EGFR) being a key target for new, effective treatments crucial for the signaling pathways regulating cancer cell survival. Targeting EGFR-mediated signaling offers promising strategies to improve NSCLC therapies, particularly in overcoming resistance in EGFR-mutant lung cancer. In this study, we investigated the anticancer effects of panduratin A, a naturally occurring flavonoid from *Boesenbergia rotunda*, on human NSCLC cell lines expressing both wild-type EGFR (A549) and mutant EGFR (H1975) using in vitro experiments and molecular docking approaches. Cytotoxicity screening revealed that panduratin A exhibits potent effects on both A549 (IC_50_ of 6.03 ± 0.21 µg/mL) and H1975 (IC_50_ of 5.58 ± 0.15 µg/mL) cell lines while demonstrating low toxicity to normal MRC5 lung cells (12.96 ± 0.36 µg/mL). Furthermore, western blotting and flow cytometric analyses indicated that panduratin A induces apoptosis by inhibiting p-EGFR and its downstream effectors, p-STAT3 and p-Akt, in lung cancer cells. Additionally, the docking study showed lower binding energy between panduratin A and the target proteins, comparable to that of epidermal growth factor receptor tyrosine kinase inhibitors (EGFR TKIs). The ADMET prediction also highlighted panduratin A’s exceptional drug-like properties. This study concludes that panduratin A shows significant promise as an anti-lung cancer candidate for NSCLC, offering an economical and effective strategy.

## 1. Introduction

Lung cancer represents a significant public health challenge worldwide [1,2]; particularly in Thailand, where it is the most prevalent cancer and has the highest mortality rate compared to other cancer types. Furthermore, the incidence of lung cancer continues to rise [3,4,5]. Lung cancer is primarily categorized into two main types: small cell lung cancer (SCLC) and non-small cell lung cancer. Among these, NSCLC accounts for approximately 85% of cases in both smokers and non-smokers [6,7], with adenocarcinoma comprising about 60% of all NSCLC cases [8]. Approximately 80% of lung cancer cases are attributed to genetic mutations [9]. Notably, mutations in the EGFR gene are frequently associated with the development of NSCLC, particularly in the adenocarcinoma subtype. The most common mutations identified include exon 19 deletions and exon 21 L858R substitutions, which together account for 85–90% of cases [10,11]. These mutations act as oncogenic drivers in NSCLC development by enhancing EGFR kinase activity and subsequently activating downstream pro-survival signaling pathways [12].

NSCLC is frequently insidious, often presenting no symptoms until the disease is significantly advanced. Approximately 80–85% of lung cancer patients are diagnosed with locally advanced or metastatic disease [13], which contributes to poor survival outcomes, as evidenced by a 5-year survival rate of less than 17.8% [14]. In cases of metastatic lung cancer, local treatments are typically ineffective, necessitating the use of chemotherapy [15]. Chemotherapy employs anticancer drugs containing chemical agents designed to eradicate cancer cells throughout the body while inhibiting their further growth and division [16]. However, this treatment modality is often accompanied by toxic side effects, as chemotherapy indiscriminately targets both malignant and normal cells, leading to unintended damage to healthy tissues. Over the past decade, cancer treatment has progressively shifted towards targeted therapy, with EGFR TKIs emerging as highly effective options for NSCLC patients harboring EGFR mutations. These agents are designed to bind to the adenosine triphosphate binding domain of the EGFR protein, thereby disrupting downstream signaling pathways [17]. Clinical studies have reported an impressive objective response rate of approximately 70% for these therapies, with progression-free survival extending to 8–13 months [12]. Furthermore, EGFR TKIs have demonstrated a significant improvement in progression-free survival compared to traditional chemotherapy. However, the emergence of the T790M mutation in the EGFR gene has been associated with therapeutic resistance, leading to the eventual failure of first- or second-generation inhibitors after 9–13 months of treatment with EGFR TKIs [18,19]. Currently, osimertinib is the most widely accepted first-line treatment option for patients with advanced NSCLC harboring EGFR mutations [17]. This agent has demonstrated improved survival outcomes compared to first-generation TKIs in the first-line setting. However, despite osimertinib’s efficacy, its high cost significantly restricts clinical use and accessibility for many patients. Previous studies have indicated that, when assessed for cost-effectiveness, osimertinib is unlikely to be regarded as a viable alternative due to its expense [20,21,22]. Additionally, these inhibitors are associated with various serious side effects that necessitate careful monitoring [23]. Furthermore, their efficacy is limited in the context of non-EGFR mutations and co-mutations; for instance, EGFR/Kirsten rat sarcoma viral oncogene homolog (KRAS) co-mutations may diminish the effectiveness of targeted EGFR therapies [10,17]. Consequently, there remains a pressing need to develop novel inhibitors and therapeutic strategies that specifically target the EGFR L858R/T790M mutations, with robust inhibitory activity and cost-efficiency to enhance accessibility for a broader patient population.

The EGFR is a multifunctional glycoprotein that is prominently expressed in both normal tissues and various cancer-affected organs. Numerous studies have demonstrated that EGFR-mediated signaling pathways are crucial for tumor cell proliferation, angiogenesis, invasion, metastasis, and apoptosis [24]. Consequently, EGFR is considered a promising therapeutic target in cancer treatment, particularly for NSCLC [25]. The key proteins regulated by EGFR, which have been extensively studied in relation to the intracellular mechanisms of NSCLC, involve three major signaling pathways: rat sarcoma virus protein (Ras)/rapidly accelerated fibrosarcoma kinase (Raf)/mitogen-activated protein kinase kinase (MEK)/extracellular signal-regulated kinase (ERK), phosphoinositide 3-kinase (PI3K)/AKT, and signal transducer and activator of transcription 3 (STAT3) [26,27,28]. Research investigating the mechanisms of action of EGFR TKIs in both EGFR wild-type and EGFR mutant NSCLC has revealed that these inhibitors function by inhibiting the intracellular phosphorylation of the EGFR protein, particularly in T790M mutant variants [29]. Therefore, targeting the EGFR is paramount for developing compounds aimed at combating NSCLC through the inhibition of intracellular phosphorylation and the subsequent reduction of activation in key signaling pathways. This research aims to identify natural compounds that exhibit high toxicity against lung cancer cells while maintaining low toxicity to normal cells.

*Boesenbergia rotunda* is a tropical plant celebrated for its dual role as a flavorful spice and a revered ingredient in traditional medicine. In vitro studies of extracts from *B. rotunda* and its isolated compounds have demonstrated a wide range of pharmacological activities. The two primary compounds present in *B. rotunda* are panduratin A and pinostrobin [30,31]. Both compounds have been reported to exhibit anti-lung cancer activity against the wild-type NSCLC cell line A549 [32,33]. Previous research has shown that panduratin A activates protective autophagy in melanoma cells via the adenosine monophosphate-activated protein kinase (AMPK) and mammalian target of rapamycin (mTOR) signaling pathways [34]. Additionally, panduratin A has been found to inhibit cell proliferation with minimal effects on normal human MCF-10A breast cells [35]. Despite the numerous reported pharmacological effects of panduratin A and pinostrobin, their anticancer activity against human NSCLC remains largely underexplored.

In the present study, we aimed to identify the most potent cytotoxic effects of panduratin A and pinostrobin on human NSCLC cell lines, specifically those expressing wild-type EGFR and G12S exon 2 KRAS (A549), as well as the L858R/T790M EGFR (H1975) and wild-type KRAS [36]. We investigated the mechanisms of cell death utilizing a variety of experimental techniques, including western blotting, flow cytometry, and molecular docking simulations. These methodologies provided valuable insights into the effects of panduratin A, enhancing our comprehension of its biological impact. Molecular docking studies were employed to elucidate the complex interactions between panduratin A and cellular components at the molecular level. This comprehensive approach significantly enriches our understanding of how panduratin A orchestrates cell death processes.

## 2. Results

### 2.1. In Vitro Cytotoxicity Screening of Pinostrobin and Panduratin A Against NSCLC Cell Lines

We conducted a screening to evaluate the cytotoxic potency of panduratin A and pinostrobin, isolated from the roots and rhizomes of *B. rotunda* using the centrifugal partition chromatography (CPC) technique, in comparison with first-generation (gefitinib) and third-generation (osimertinib) EGFR TKIs. The compounds were tested against NSCLC cell lines expressing wild-type EGFR (A549) and the mutant L858R/T790M EGFR (H1975). Cytotoxicity was assessed using the MTT assay (3-(4,5-dimethylthiazol-2-yl)-2,5-diphenyltetrazolium bromide assay). According to the United States National Cancer Institute’s classification, cytotoxicity is categorized based on the half-maximal inhibitory concentration (IC_50_) value as follows: IC_50_ < 20 µg/mL indicates high cytotoxic activity, IC_50_ of 21–200 µg/mL indicates moderate cytotoxic activity, IC_50_ of 201–500 µg/mL indicates weak cytotoxic activity, and IC_50_ > 500 µg/mL indicates no cytotoxic activity [37,38].

**Figure 1:**
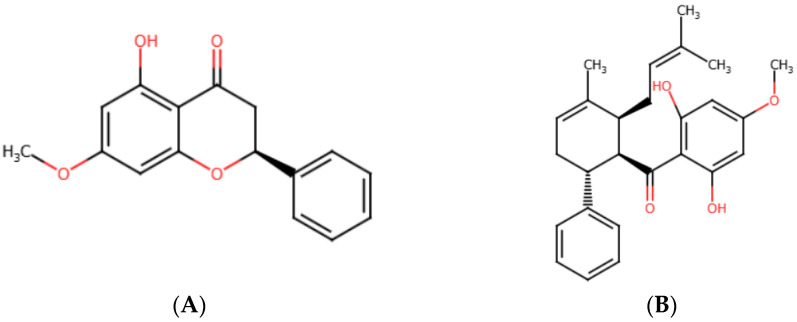
Chemical structures of (**A**) pinostrobin and (**B**) panduratin A.

The data presented in Table 1 indicated that the IC_50_ value of pinostrobin could not be determined due to its poor solubility in aqueous media. Crystalline formations were observed in the cell culture medium of both A549 and H1975 cells after treatment with pinostrobin for 24 h (see Figure S1 in Supplementary Material). The molecular structure of pinostrobin (Figure 1A) features intramolecular hydrogen bonding, with hydroxyl (OH) groups interacting with nearby carbonyl (C=O) groups. This structural arrangement diminishes its capacity to form hydrogen bonds with water, resulting in limited solubility. Consequently, this poor solubility restricts pinostrobin’s ability to effectively interact with cell membranes and exert its therapeutic effects [38,39]. Therefore, it is recommended that pinostrobin be formulated into inclusion complexes to enhance its solubility, thereby improving its potential as a potent anticancer agent. The structure of panduratin A (Figure 1B) indicates that it has better solubility in aqueous media compared to pinostrobin. This is due to the presence of two OH groups in panduratin A, which are considered hydrophilic, as they form hydrogen bonds with water and enhance solubility.

**Figure 2:**
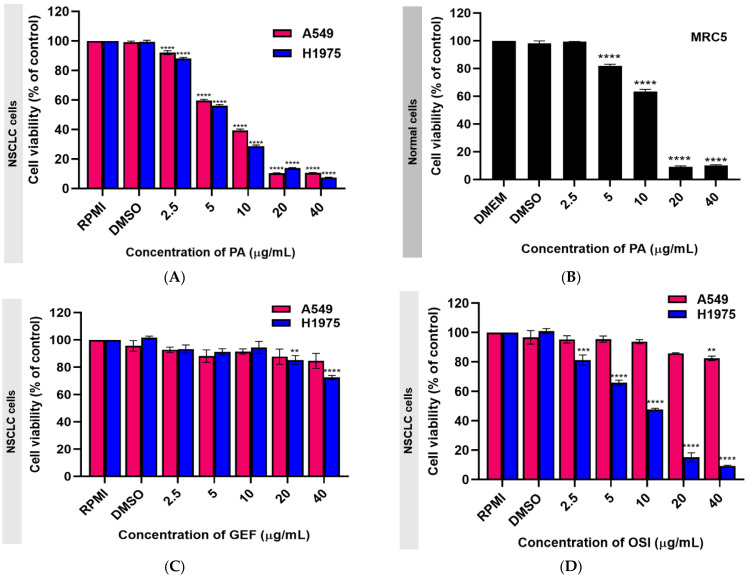
Cell viability of (**A**,**C**,**D**) A549, H1975, and (**B**) MRC5 cell lines after treatment with various concentrations of panduratin A (PA), gefitinib (GEF), and osimertinib (OSI) for 24 h. Data are expressed as the mean ± SEM of three independent experiments. ** *p* ≤ 0.01, *** *p* ≤ 0.001, and **** *p* ≤ 0.0001 vs. control.

In Table 1, the IC_50_ values from the cytotoxicity study of panduratin A on A549, H1975, and normal MRC5 cells are reported. Meanwhile, Figure 2 illustrates the effect of various panduratin A concentrations on cell viability. This study revealed that panduratin A exhibited cytotoxic effects against both A549 and H1975 cells, inhibiting cell survival in a concentration-dependent manner. The IC_50_ values were determined to be 6.03 ± 0.21 µg/mL for A549 cells and 5.58 ± 0.15 µg/mL for H1975 cells. Furthermore, panduratin A demonstrated a greater toxicity to cancer cells compared to normal lung cells, which had an IC_50_ value of 12.96 ± 0.36 µg/mL. When considering the selectivity index (SI), panduratin A has exhibited an SI greater than two in both A549 and H1975 cells, indicating selective toxicity toward lung cancer cells compared to normal lung cells. Additionally, panduratin A demonstrated more potent cytotoxicity than gefitinib (IC_50_ > 40 µg/mL for both A549 and H1975 cells) and osimertinib (IC_50_ > 40 µg/mL for A549 and 9.466 ± 0.307 µg/mL for H1975), which are commonly employed targeted therapies for NSCLC patients, as evidenced by the lower IC_50_ values associated with panduratin A.

**Figure 3:**
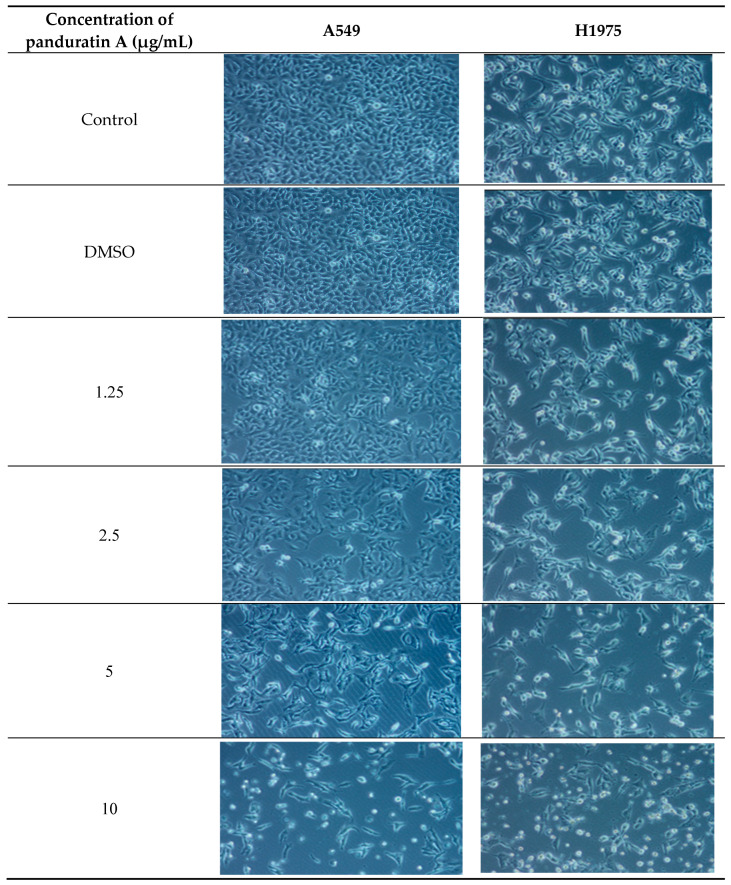
Morphological changes in A549 and H1975 cell lines after 24 h treatment with various concentrations of panduratin A. Microscope images were taken at 4X.

This section of the experiment aims to highlight the limitations of EGFR-TKIs in terms of treatment efficacy. As shown in Figure 2D, osimertinib inhibits H1975 cells expressing the L858R/T790M EGFR mutation but demonstrates minimal activity against the wild-type EGFR. These findings are consistent with previous reports [40,41]. Gefitinib also displayed low toxicity against both A549 and H1975 cells (Figure 2C), which can be attributed to its limited efficacy in the presence of non-EGFR mutations [42,43,44]. Additionally, the morphological changes observed in A549 and H1975 NSCLC cells after 24 h of treatment with panduratin A, illustrated in Figure 3, indicate that panduratin A induces dose-dependent cellular shrinkage, a characteristic associated with programmed cell death [2]. This suggests that panduratin A promotes cell death through an apoptotic mechanism.

The results indicate that panduratin A possesses a higher cytotoxic potential against A549 and H1975 cells compared to the standard drugs used for NSCLC. Consequently, panduratin A was selected for further investigation to elucidate the mechanisms underlying panduratin A-induced cell death in NSCLC cell lines, utilizing both experimental and theoretical approaches.

### 2.2. Panduratin A Induces Apoptosis in A549 and H1975 Cell Lines

**Figure 4:**
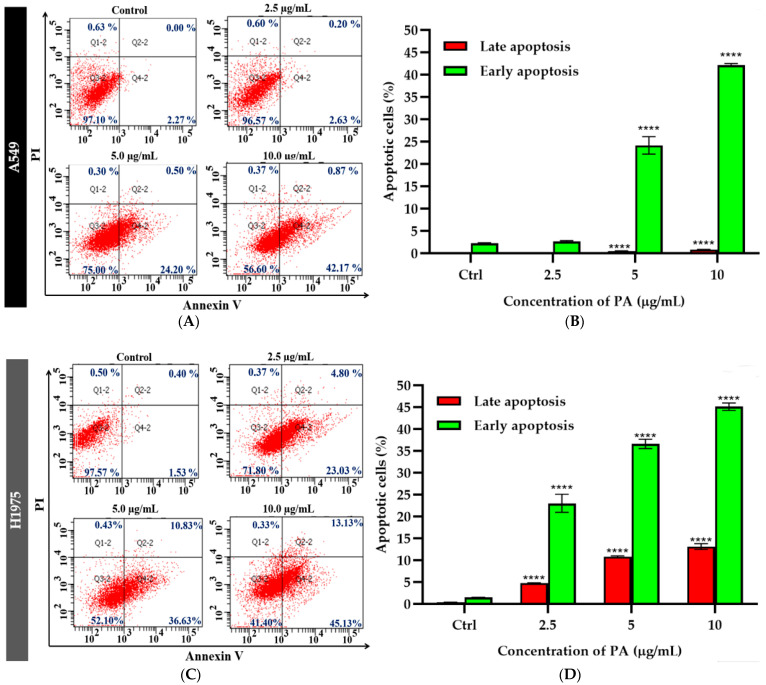
Flow cytometric analysis of Annexin V/PI-stained cells after panduratin A (PA) treatment for 24 h on A549 (**A**,**B**) and H1975 (**C**,**D**) cells. Representative figures showing populations of viable (Q3), early apoptotic (Q4), late apoptotic (Q2), and necrotic (Q1) cells. The red dot plot displaying the cell population by fluorescence intensity. Data are expressed as the mean ± SEM of three independent experiments. **** *p* ≤ 0.0001 vs. control.

To investigate whether apoptotic mechanisms are involved in the panduratin A-induced cytotoxicity in A549 and H1975 cells, a flow cytometric analysis of Annexin V/PI-stained cells was performed. After 24 h of treatment, panduratin A at a concentration of 5 µg/mL significantly induced apoptotic cell death in the A549 cells, whereas panduratin A at 2.5 µg/mL significantly induced apoptotic cell death in the H1975 cells (Figure 4). These results suggest that the apoptosis-inducing effect of panduratin A is more pronounced in NSCLC cells harboring the mutant EGFR than in cells with the wild-type EGFR. Interestingly, the treatment with panduratin A in H1975 cells led to a striking, dose-dependent increase in apoptosis, highlighting panduratin A’s potent cytotoxic effects.

These results provide compelling evidence that panduratin A exerts its cytotoxic effects in A549 and H1975 cells through the induction of apoptosis, which is consistent with previous studies [32,45]. This study is the first to report the significant cytotoxic effect of panduratin A on the H1975 lung cancer cell line, an established model bearing the T790M mutation in addition to the L858R mutation, which is commonly used to study acquired resistance to EGFR-TKIs [46,47]. Furthermore, the dose-dependent nature of H1975 cell death provides valuable insights for developing effective and safe therapies to overcome EGFR-TKI resistance in future cancer treatments.

### 2.3. Panduratin A Inhibits EGFR, STAT3, and Akt Signaling Pathways in A549 and H1975 NSCLC Cell Lines

**Figure 5:**
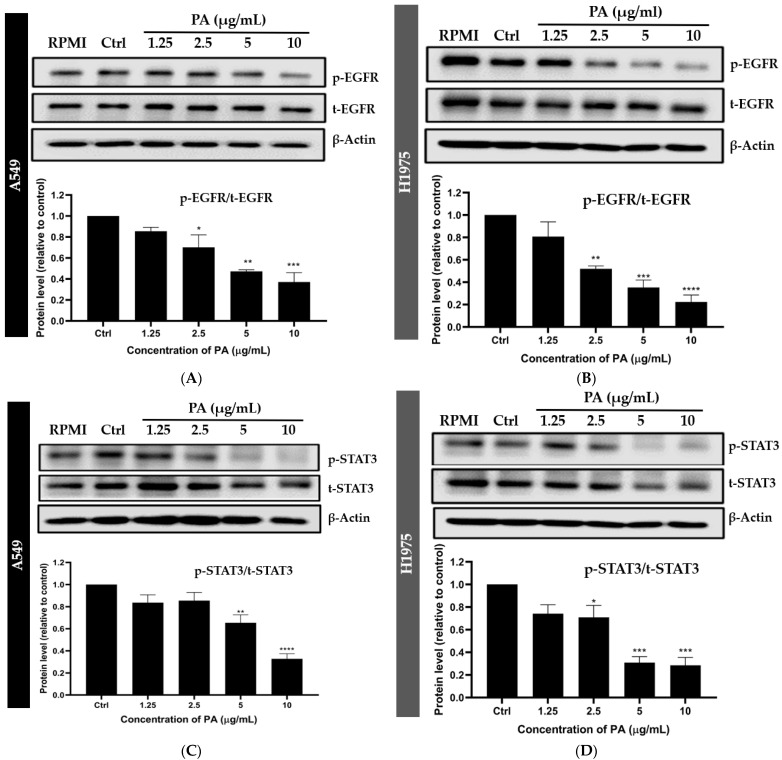

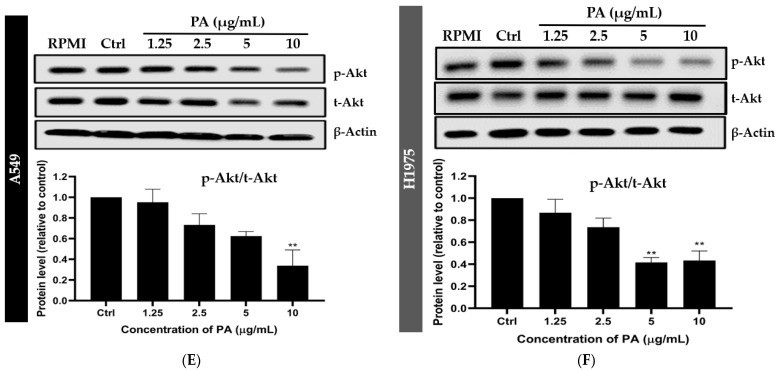
Panduratin A (PA) inhibits the phosphorylation of EGFR (**A**,**B**), STAT3 (**C**,**D**), and Akt (**E**,**F**) in A549 and H1975 cells at 24 h. Data are expressed as the mean ± SEM of three independent experiments. * *p* ≤ 0.05, ** *p* ≤ 0.01, *** *p* ≤ 0.001, and **** *p* ≤ 0.0001 vs. control.

To elucidate the effects of panduratin A on EGFR-mediated survival signaling pathways, western blot analysis was conducted. As depicted in Figure 5, panduratin A significantly inhibited the phosphorylation of the EGFR (Figure 5A,B), STAT3 (Figure 5C,D), and Akt (Figure 5E,F) in both A549 and H1975 cells. Notably, panduratin A exhibited the concentration-dependent inhibition of the EGFR-mediated survival signaling pathway in H1975 cells compared to A549 cells. This finding suggests that panduratin A may preferentially target the EGFR T790M mutation, which is a critical focus for therapeutic strategies aimed at overcoming drug resistance in NSCLC, as reported in previous studies [48].

**Figure 6:**
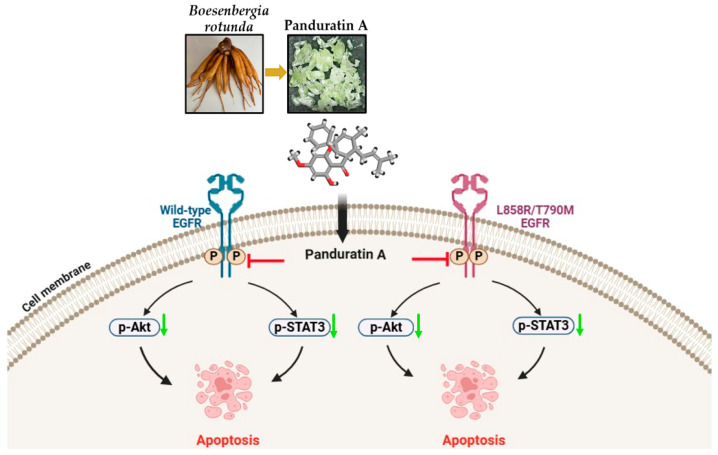
Mechanisms of panduratin A (PA) against A549 and H1975 NSCLC cell lines, in which panduratin A promotes cell apoptosis through the inhibition of p-EGFR, p-Akt, and p-STAT3 signaling pathways. The black arrow indicates activation, the red arrow indicates inhibition, and the green down arrow indicates down-regulation of the protein.

Figure 6 illustrates the proposed underlying mechanisms through which panduratin A affects the two studied NSCLC cell lines, A549 and H1975. Specifically, panduratin A promotes cell apoptosis by inhibiting the phosphorylation of the EGFR, Akt, and STAT3 signaling pathways. This study represents the first discovery of novel treatment pathways for NSCLC involving panduratin A, which demonstrates mechanisms similar to those of osimertinib in inhibiting EGFR phosphorylation in cell lines harboring sensitizing EGFR mutations as well as in T790M mutant cell lines [29,49].

### 2.4. Predictive Binding Affinity of Panduratin A Against EGFR, STAT3, and Akt Signaling Proteins

Given that we observed that panduratin A inhibits p-EGFR, p-STAT3, and p-Akt, we further investigated the atomistic binding mechanisms of panduratin A against these target proteins, leading to phosphorylation inhibition. This was accomplished using molecular docking techniques to estimate the binding affinity of panduratin A against the key proteins EGFR, STAT3, and Akt, in comparison with known inhibitors, such as osimertinib and gefitinib. To assess the accuracy of the molecular docking parameters and methods in replicating natural binding poses, a redocking experiment was conducted using co-crystallized compounds. The root mean square deviation (RMSD) values ranged from 1.350 to 1.894 Å (with an acceptable RMSD threshold set at ≤2.0 Å) [50], confirming that the docking method and parameters employed reliably predicted the native conformations of the compounds.

**Figure 7:**
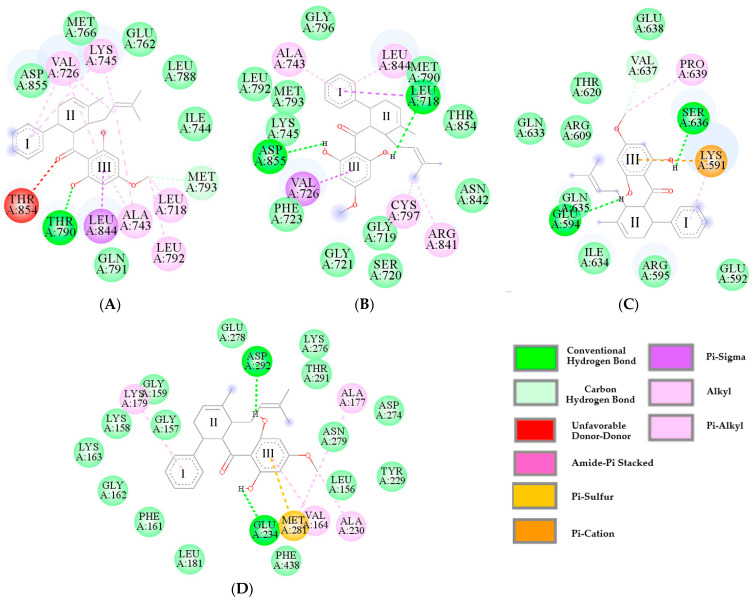
Binding interactions of panduratin A with (**A**) EGFR^WT^ (7UKV), (**B**) EGFR^T790M^ (5Y9T), (**C**) STAT3 (1BG1), and (**D**) Akt (4GV1).

The results presented in Table 2 indicate that the values of ΔG_bind_ and inhibition constant (Ki) were correlated. Specifically, lower values of ΔG_bind_ were associated with lower values of Ki. It was noted that the ΔG_bind_ values obtained for all complexes were very similar, with negligible differences between them. On the other hand, the difference between Ki values of all the complexes were significant. Overall, the results predicting the Ki were ranked in the order of osimertinib < panduratin A < gefitinib < pinostrobin. Based on the Ki values, it can be concluded that panduratin A had a better inhibitory potency against cancer than pinostrobin and gefitinib. However, its effectiveness was still approximately half that of osimertinib when targeting EGFR^WT^, EGFR^T790M^, and Akt, while it showed a higher Ki value than osimertinib for STAT3. The docking study revealed that panduratin A interacted with the protein crystal structures via key residues, as illustrated in Figure 7 and detailed in Table 3. Notably, panduratin A bound to the protein targets through two major types of interactions: hydrogen bonds and hydrophobic interactions. The aromatic, methoxy, and hydroxyl functional groups (Ring III) of the ligand served as important structural sites for bond formation with the residues, consistent with findings from previous studies [51]. Therefore, the obtained ΔG_bind_ score for panduratin A indicates a binding affinity for the protein/inhibitor complex that is comparable to that of osimertinib. Both the theoretical and experimental findings suggest that the anticancer activity of panduratin A arises from its capacity to bind to the EGFR^WT^, EGFR^T790M^, STAT3, and Akt signaling proteins. These interactions play a crucial role in inhibiting signaling pathways that induce apoptotic processes, which align well with the observed trends in the experimental results.

### 2.5. Pharmacokinetic Characteristics and ADMET Prediction

**Figure 8:**
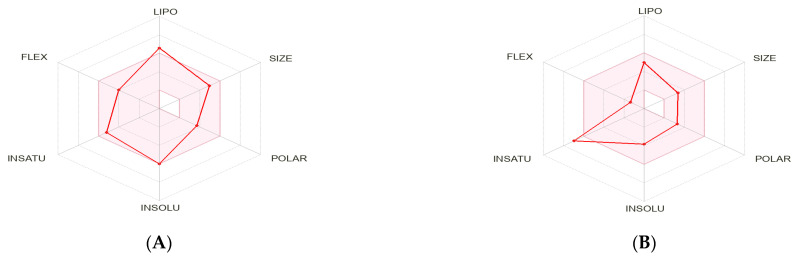

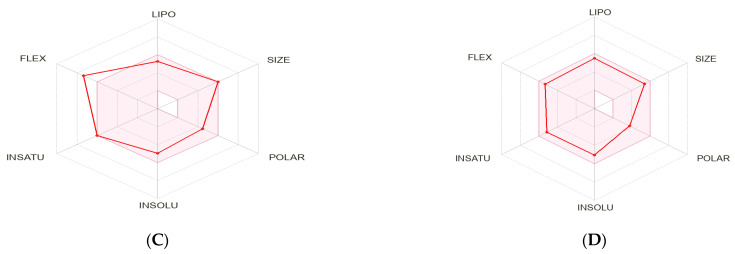
Pink zoned oral bioavailability radars for (**A**) panduratin A (PA), (**B**) pinostrobin (PN), (**C**) osimertinib (OSI), and (**D**) gefitinib (GEF).

To further investigate the potential of panduratin A and pinostrobin as orally bioavailable candidates, compared to commercial EGFR-targeted drug scaffolds, we evaluated their absorption, distribution, metabolism, and excretion (ADME) properties using the Swiss ADME webserver [52] and admetSAR [53] free online tool. As shown in Table 4, panduratin A demonstrates favorable drug-like properties, fully adhering to Veber’s Rule with no violations. Although there is one violation of Lipinski’s Rule of Five (specifically, the LogP value, which should be below 5), it is important to note that the rule typically allows for a single violation in orally active drugs [54]. This suggests that, despite this minor deviation, panduratin A may still be a promising candidate for further development as an oral therapeutic agent. This indicates that panduratin A possesses acceptable properties comparable to those of the reference drugs gefitinib and osimertinib, as established by the outlined criteria [55]. Additionally, the toxicity analysis confirmed that both compounds are non-mutagenic and non-tumorigenic. The bioavailability profile for scaffolds containing panduratin A is illustrated in Figure 8. This radar plot features six axes that represent six essential characteristics of oral bioavailability: solubility (INSOLU), flexibility (FLEX), size (SIZE), lipophilicity (LIPO), saturation (INSATU), and polarity (POLAR). The pink region in the radar plot indicates the optimal property parameters for oral bioavailability, while the red lines represent the scaffolds of the compounds. Panduratin A is located within the pink area, suggesting that the designed scaffolds have an acceptable estimated oral bioavailability.

**Figure 9:**
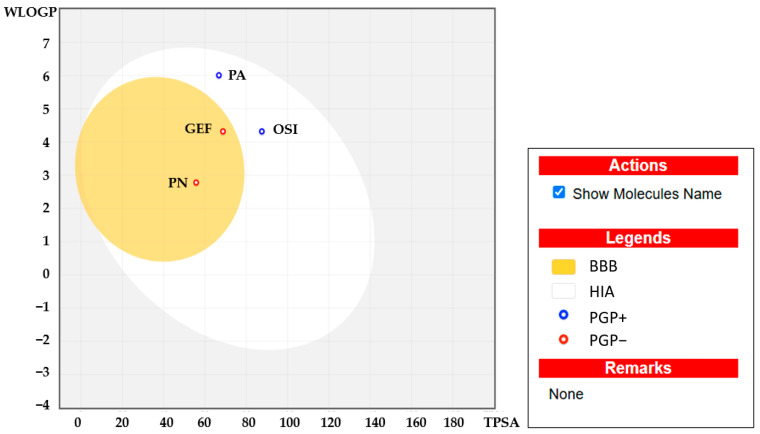
Boiled Egg plot showing water partition coefficient (WlogP) vs. topological polar surface area (TPSA) of panduratin A (PA) and pinostrobin (PN) compared with reference drugs osimertinib (OSI) and gefitinib (GEF).

Figure 8 provides further insight into the lipophilicity of panduratin A, with a log P value of 6.01, which falls within the acceptable range of −2 to 6.5. Computational bioavailability properties assessed through a Boiled Egg analysis (WlogP vs. TPSA) indicated that both panduratin A and pinostrobin have high absorption potential from the intestinal tract following oral administration (high intestinal absorption is acceptable if >80%). This suggests that panduratin A is unlikely to have negative effects on the central nervous system due to its low blood–brain barrier permeability (depicted in white in Figure 9) and the active efflux from the central nervous system or gastrointestinal lumen mediated by P-glycoprotein (PGP+) (illustrated by the blue dot). Furthermore, the ADMET prediction analysis revealed that pinostrobin also demonstrates excellent oral bioavailability and is a non-substrate for P-glycoprotein (indicated by the blue dot) [51].

## 3. Discussion

Chemotherapy and first-generation TKIs have been employed as first-line treatments for NSCLC patients with wild-type and mutant EGFRs, respectively. However, conventional chemotherapeutic agents lack selectivity for cancer cells, often exhibiting a higher cytotoxicity toward normal cells [56]. Additionally, the development of acquired drug resistance, particularly due to the T790M mutation in the EGFR gene, is a significant concern. Indeed, all patients with EGFR-mutant NSCLC will inevitably develop acquired resistance after a period of treatment with EGFR TKIs [57]. Consequently, cancer therapy is constrained by the toxicity to normal cells and the emergence of drug resistance in cancer cells, highlighting an urgent need for novel anticancer compounds effective against both wild-type and T790M-positive EGFR-expressing NSCLC.

Our findings indicated that panduratin A, isolated from *B. rotunda*, exhibits significant anti-lung cancer activity and presents a promising therapeutic option for NSCLC patients with both the wild-type and mutant EGFR, while also demonstrating low toxicity to MRC5 normal cells. This research was the first to investigate the EGFR/STAT3/Akt signaling pathways in both wild-type and T790M mutations in the EGFR mechanism of panduratin A. We elucidated the cytotoxic activity of panduratin A, both experimentally and theoretically, revealing potent cytotoxic effects toward A549 and H1975 cells. Notably, panduratin A has demonstrated a greater cytotoxicity than gefitinib against both A549 and H1975 cell and was more effective than osimertinib in A549 cells, possibly due to the limited efficacy of osimertinib in non-EGFR mutations [42,44]. In this study, while panduratin A exhibited a higher cytotoxicity than osimertinib in H1975 cells, previous research has highlighted that osimertinib has a significantly lower SI in MRC5 normal cells [58] compared to panduratin A, which is consistent with our findings. Conversely, the SIs of gefitinib (SI = 0.81) and osimertinib (SI = 1.15) concerning A549 NSCLC cells and normal human bronchial epithelial cells are considerably lower than that of panduratin A [59]. It is important to note that this study evaluated only one normal cell line; consequently, further investigations involving additional normal cell lines are necessary to comprehensively assess the selectivity and efficacy of panduratin A.

The ADMET prediction results indicated that pinostrobin has demonstrated excellent oral bioavailability and several potentially beneficial biological activities. However, its low solubility in aqueous media presents a significant challenge for pharmaceutical applications. Our findings align with previous studies that suggest pinostrobin’s reduced toxicity is attributable to its low solubility [39]. Consequently, pinostrobin was excluded from our mechanistic studies. Further research is necessary to enhance its solubility prior to conducting cytotoxicity studies. Therefore, panduratin A was selected for further investigation into the mechanisms underlying cell death.

Previous research has established that the primary cause of NSCLC is often mutations in the EGFR gene, which result in excessive intracellular signaling and uncontrolled cancer cell proliferation, as well as the development of resistance to EGFR-targeted therapies [18,19]. Consequently, the EGFR serves as a crucial biomarker and therapeutic target for the treatment of NSCLC. Three key signaling pathways regulated by EGFR—Ras/Raf/MEK/ERK, PI3K/AKT, and STAT3—are essential for understanding the mechanisms underlying NSCLC. EGFR TKIs, such as gefitinib and osimertinib, act by inhibiting the phosphorylation of the EGFR within cells, thereby targeting these pathways. Numerous studies have demonstrated the presence of bioactive phytochemicals in extracts from *B. rotunda*, including the chalcone derivatives panduratin A and pinostrobin. This study confirmed that all tested ligands (panduratin A, pinostrobin, and the reference drugs gefitinib and osimertinib) could interact with critical residues in the catalytic sites of all targeted proteins. The obtained ΔG_bind_ score for panduratin A indicates a strong binding affinity for the protein/inhibitor complex, comparable to that of osimertinib. Additionally, the ADMET prediction results revealed that panduratin A exhibits excellent oral bioavailability compared to the reference drugs. These findings have prompted us to conduct further investigations in subsequent in vitro studies.

In this study, western blotting and flow cytometric analyses indicated that panduratin A induces apoptosis by inhibiting phosphorylated EGFR (p-EGFR) and its downstream effectors, phosphorylated STAT3 (p-STAT3) and phosphorylated Akt (p-Akt), in both A549 and H1975 lung cancer cell lines. We elucidated the mechanisms of phosphorylation involving EGFR, STAT3, and Akt through the docking analysis of panduratin A. The obtained results revealed a ΔG_bind_ score for panduratin A ranging from −6.91 to −8.06 kcal/mol, indicating a strong binding affinity for the protein/inhibitor complex. Our in vitro experiments and theoretical analyses of panduratin A were correlated, consistent with previous studies demonstrating that panduratin A can inhibit the survival of PANC-1 cells under nutrient-deprived conditions through the inhibition of the PI3K/Akt/mTOR/autophagy signaling pathway [60]. Additionally, chalcone derivatives, including panduratin A, have been shown to inhibit the STAT3 pathway by interfering with the phosphorylation of STAT3 proteins and inhibiting kinase activities, such as those of janus kinase (JAK) and Src, which also impact EGFR signaling pathways [61]. In vitro EGFR kinase assays demonstrated that both chalcone derivatives and osimertinib significantly suppressed the activity of activating mutant EGFRs, including EGFR Del E746–A750, EGFR L858R/T790M, and EGFR L858R [62].

Overall, our present study provides initial insights into the underlying mechanisms of panduratin A’s effect on EGFR-mediated signaling pathways in NSCLC cell lines expressing wild-type and mutant EGFRs. However, further studies exploring additional signaling pathways are essential to gain a deeper understanding of the inhibitory effects of panduratin A on signal transduction.

## 4. Materials and Methods

### 4.1. Chemical Reagents and Antibodies

Pinostrobin and panduratin A were extracted from *B. rotunda* using ultrasound assisted (Bandelin DT1028H, Berlin, Germany) extraction with ethanol pre-treatment, followed by centrifugal partition chromatography (CPC) (model: CPC250, Gilson, Madison, WI, USA) purification according to the previous study [63]. Bovine serum albumin (BSA), dimethyl sulfoxide (DMSO), gefitinib, MTT, and protease inhibitors were purchased from Sigma-Aldrich (St. Louis, MO, USA). Osimertinib was obtained from Cayman Chemical (Ann Arbor, MI, USA). RIPA lysis buffer was obtained from Thermo Fisher Scientific (Waltham, MA, USA). Protein assay reagents were sourced from Bio-Rad (Hercules, CA, USA). Antibodies against phospho-EGFR (p-EGFR, 2234), total-EGFR (t-EGFR, 4267), phospho-STAT3 (p-STAT3, 9145), total-STAT3 (t-STAT3, 12640), phospho-Akt (p-Akt, 4060), total-Akt (t-Akt, 4691), Beta Actin rabbit monoclonal antibody (β-actin, 8457), and anti-rabbit IgG HRP-linked antibody (7074) were purchased from Cell Signaling Technology (Santa Cruz, CA, USA).

### 4.2. Cell Lines and Culture

Human NSCLC cell lines A549 and H1975, as well as human normal lung cell line (MRC5), were purchased from American Type Culture Collection (ATCC, Manassas, VA, USA). A549 cells and H1975 cells were cultured in RPMI-1640 medium containing 10% FBS, 100 U/mL penicillin, and 100 g/mL streptomycin. The DMEM with high glucose (4500 mg/L) supplemented with 10% FBS, 100 U/mL penicillin, and 100 g/mL streptomycin was used for culturing MRC5 cells. All cells were maintained at 37 °C in a humidified 5% CO_2_ atmosphere.

### 4.3. Cell Viability Assay

Cell viability was assessed using the MTT assay. Cells were seeded into 96-well plates at a density of 5 × 10^3^ cells/well for H1975 and MRC5, and 3 × 10^3^ cells/well for A549. After overnight incubation, cells were treated with panduratin A, gefitinib, and osimertinib at concentrations of 2.5, 5, 10, 20, and 40 µg/mL for 24 h, and pinostrobin at concentrations of 6.25, 12.5, 25, 50, and 100 µg/mL for 24 h. Subsequently, MTT solution (5 mg/mL) was added and incubated for 4 h. The medium was then removed, and 150 µL of DMSO was added to each well. Finally, the absorbance of the formazan product was measured at a wavelength of 570 nm using a LabSystems Multiskan MS microplate reader (Thermo Scientific, Vantaa, Finland). The Selectivity Index (SI) was calculated as follows: SI = IC_50_ of a compound in a normal cell line/IC_50_ of the same compound in a cancer cell line. SI values greater than 2 were considered indicative of acceptable selectivity [64].

### 4.4. Western Blotting

A549 and H1975 cells were seeded into 6-well plates at a density of 2 × 10^5^ cells/well and 3 × 10^5^ cells/well, respectively. After overnight incubation, the cells were treated with the panduratin A at concentrations of 1.25, 2.5, 5, and 10 µg/mL. After 24 h of incubation, the cells were rinsed three times with cold PBS, homogenized in RIPA buffer containing a protease inhibitor, and incubated at 4 °C for 15 min. The samples were then centrifuged at 18,620 RCF (4 °C) for 15 min. The protein content was determined by the Lowry method using bovine serum albumin as a standard. Total protein (20 µg) was separated by 10% SDS-PAGE and subsequently transferred to a PVDF membrane. The membrane was blocked with 5% non-fat dry milk for 1 h, then incubated with the primary antibody at 4 °C overnight. After incubation, the membrane was washed three times with TBST buffer (5 min each) and incubated with HRP-linked secondary antibody for 2 h at room temperature. Immunoreactive bands were detected using HRP substrate (Millipore, Billerica, MA, USA) and quantitatively measured using Image Studio Lite software (version 5.2.5, LI-COR, Lincoln, NE, USA). β-Actin was used as an internal control for protein normalization.

### 4.5. Flow Cytometric Evaluation of Apoptosis

NSCLC cells were plated onto 6-well plates at a density of 2 × 10^5^ cells/well for A549 cells and 3 × 10^5^ cells/well for H1975 cells. After overnight incubation, the cells were treated with 2.5, 5, and 10 µg/mL panduratin A for 24 h. After treatment, dead or non-adhered cells were collected, while adherent cells were harvested by trypsinization. The cells were then washed with PBS (pH 7.4) and centrifuged at 750 RCF at 4 °C for 5 min. Subsequently, the cells were resuspended in Annexin V binding buffer and stained with 5 µL of Annexin V fluorescein dye and 2 µL of propidium iodide (PI) at room temperature for 20 min in the dark. Following staining, the cells were resuspended in 400 µL of cold Annexin V binding buffer prior to flow cytometry analysis. The cells were analyzed using a BD FACSAria™ II flow cytometer (BD Biosciences, Franklin Lakes, NJ, USA). Data analysis was performed using FlowJo™ software (version 9.9.3; Tree Star, Inc., Ashland, OR, USA).

### 4.6. Molecular Docking

The crystal structures of the human EGFR wild type (PDB ID: 7UKV) [65], EGFR mutant type (PDB ID: 5Y9T) [66], STAT3 (PDB ID: 1BG1) [2], and Akt1 (PDB ID: 4GV1) [2] were obtained from Protein Data Bank (PDB). Preparation of target proteins and ligands were replicated from the previous study [50]. The protein–ligand complexes were generated using AutoDock 4.2.6 with 50 docking runs and a grid point configuration of 50 × 50 × 50. Target proteins and ligands were prepared using Discovery Studio Visualizer to separate the co-crystallized ligands from their respective macromolecules, and the data were stored in .pdb file format. A re-docking experiment was performed utilizing the co-crystal compound to verify whether the molecular docking parameters and methods could accurately replicate the natural binding poses. This involved separating the ligands, which were then docked with their corresponding targets to determine the most appropriate docking poses. The grid parameters that resulted in the lowest RMSD values (less than 2 Å) were selected to study the interactions between compounds and proteins, ensuring that the docking results were accurate and reliable. Each compound was docked using the same parameters as the native ligand. The most favorable binding poses of the compounds were explored by evaluating the binding free energy (ΔG) and the estimated inhibition constant (Ki).

### 4.7. Assessment of Drug-Likeness and In Silico ADMET Prediction

Drug-likeness and the ADMET profiles were replicated from the previous study [52,53] using admet structure–activity relationship (admetSAR) 2.0 tool/database http://lmmd.ecust.edu.cn/admetsar2/ (accessed on 1 January 2025) and an online version of SwissADME web tool http://www.swissadme.ch (accessed on 15 January 2025). For this analysis, the Simplified Molecular Input Line Entry System (SMILES) formats of all the ligands were obtained from PubChem database.

### 4.8. Statistical Analysis

The quantitative data are expressed as the mean ± standard error of the mean from triplicate experiments. Differences between groups were assessed using one-way analysis of variance (ANOVA), followed by a Tukey post hoc test. Differences were considered statistically significant at *p* < 0.05.

## 5. Conclusions

This study represents the first investigation into the interplay of the EGFR, STAT3, and Akt signaling pathways within the context of panduratin A’s mechanism of action, specifically examining both the wild-type and T790M mutant forms of EGFR. The experimental and theoretical results from this study provide valuable insights into the anticancer activity of panduratin A derived from *B. rotunda*, along with its mechanisms of action against human NSCLC cell lines expressing both wild-type (A549) and mutant EGFRs (H1975). These findings suggest the potential of panduratin A as a candidate for the treatment of lung cancer, offering potent inhibitory effects, relative safety, and cost-efficiency, which could enhance accessibility for a larger patient population. Furthermore, these results establish a theoretical foundation for the development of new compounds targeting the EGFR, STAT3, and Akt signaling pathways.

## Figures and Tables

**Table 1:** IC_50_ values and selectivity index of compounds against A549 and H1975 NSCLC cells and MRC5 normal cells, determined following treatment with various doses of compounds for 24 h.

Compounds	IC_50_ (µg/mL)			Selectivity Index	
	A549	H1975	MRC5	A549	H1975
Panduratin A	6.030 ± 0.21	5.578 ± 0.15	12.963 ± 0.36	2.15	2.32
Pinostrobin	>100, due to the compound’s poor solubility in RPMI medium		_	_	_
Gefitinib	>40	>40	_	_	_
Osimertinib	>40	9.466 ± 0.307	_	_	_

**Table 2:** Molecular docking results for panduratin A, pinostrobin, and reference drug at the main binding site of EGFR^WT^, EGFR^T790M^, STAT3, and Akt.

Compounds	Protein Targets							
	EGFR^WT^ (7UKV)		EGFR^T790M^ (5Y9T)		STAT3 (1BG1)		Akt (4GV1)	
	Ki (µM)	ΔG_bind_ Score (kcal/mol)	Ki (µM)	ΔG_bind_ Score (kcal/mol)	Ki (µM)	ΔG_bind_ Score (kcal/mol)	Ki (µM)	ΔG_bind_ Score (kcal/mol)
Panduratin A	4.16	−7.34	8.68	−6.91	3.97	−7.37	1.23	−8.06
Pinostrobin	14.49	−6.60	36.94	−6.05	28.83	−6.19	1.74	−7.86
Gefitinib	6.98	−7.03	11.74	−6.73	11.35	−6.75	3.13	−7.51
Osimertinib	2.08	−7.75	3.38	−7.46	6.26	−7.10	0.439	−8.57
RMSD (redocked)	1.894		1.846		1.350		1.339	

**Table 3:** Molecular interactions between functional groups of panduratin A and protein targets.

Functional Groups	Interacting Amino Acids			
	EGFR^WT^ (7UKV)	EGFR^T790M^ (5Y9T)	STAT3 (1BG1)	Akt (4GV1)
Aromatic (Ring I)	VAL726, LYS745	LEU844, ALA743, LEU718	LYS591	LYS179
Methyl cyclohexane (Ring II)	-	CYS797, ARG841	-	-
2-methyl-2-butene	LYS745, VAL76, ALA743	-	-	-
Carbonyl	THR854	-	-	-
Aromatic (Ring III)	LEU844, ALA743, VAL726	VAL726	LYS591	MET281, VAL164
Methoxy (Ring III)	LEU718, LEU792, MET793	-	VAL637 PRO639	ALA230, MET281, ALA177
Hydroxyl (Ring III) (Hydrogen bond interactions)	THR790	ASP855, LEU718	GLU594, SER636	ASP292, GLU234

**Table 4:** ADMET outcomes of panduratin A, pinostrobin, and reference drug via Swiss ADME webserver and admetSAR.

Compounds	MW (Da)	HBD	HBA	WLogP	TPSA	RB	MR	Lipinski’s Rule of Five	Veber’s Rule	HIA (%)	BBB	Toxicity/ Carcinogenicity/ Mutagenicity
Panduratin A	406.51	2	4	6.01	66.76	6	121.48	Yes (4/5)	Yes	High 96.9%	No	No
Pinostrobin	270.28	1	4	2.78	55.76	2	74.02	Yes (5/5)	Yes	High 99.2%	Yes	No
Gefitinib	499.61	2	5	4.32	87.55	11	150.43	Yes (5/5)	No	High 94.9%	No	No
Osimertinib	446.90	1	7	4.32	68.74	8	121.66	Yes (5/5)	Yes	High 98.5%	Yes	No

Note: molecular weight (MW) ≤ 500, hydrogen bond donors (HBD) ≤ 5, hydrogen bond acceptors (HBA) ≤ 10, rotatable bond count (RB) ≤ 10, molar refractivity (MR) between 40 and 130, LogP ≤ 5, topological polar surface area (TPSA) ≤ 140 Å, high intestinal absorption (HIA) > 80%, and blood–brain barrier (BBB).

## Data Availability

Data are contained within the article and Supplementary Materials.

## Supplementary Materials

The following supporting information can be downloaded at: https://www.mdpi.com/article/10.3390/ijms26052350/s1.

## Abbreviations

The following abbreviations are used in this manuscript:

AdmetSAR	Admet structure activity relationship
AMPK	Adenosine monophosphate-activated protein kinase
ANOVA	One-way analysis of variance
ALA	Alanine
ARG	Arginine
ASP	Aspartic acid
BBB	Blood–brain barrier
BSA	Bovine serum albumin
CPC	Centrifugal partition chromatography
CYS	Cysteine
DMSO	Dimethyl sulfoxide
EGFR TKIs	Epidermal growth factor receptor tyrosine kinase inhibitors
ERK	Extracellular signal-regulated kinase
FLEX	Flexibility
GEF	Gefitinib
GLU	Glutamic acid
ΔG	Binding free energy
HIA	High intestinal absorption
IC_50_	Half-maximal inhibitory concentration
INSATU	Saturation
INSOLU	Solubility
JAK	Janus kinase
KRAS	Kirsten rat sarcoma viral oncogene homolog
Ki	Inhibition constant
LEU	Leucine
LIPO	Lipophilicity
LYS	Lysine
MEK	Mitogen-activated protein kinase
MET	Methionine
MTT	3-(4,5-dimethylthiazol-2-yl)-2,5-diphenyltetrazolium bromide
mTOR	Mammalian target of rapamycin
NSCLC	Non-small cell lung cancer
OSI	Osimertinib
PA	Panduratin A
PDB	Protein Data Bank
PI	Propidium iodide
PI3K	Phosphoinositide 3-kinase
PN	Pinostrobin
POLAR	Polarity
PRO	Proline
Raf	Rapidly accelerated fibrosarcoma kinase
Ras	Rat sarcoma virus protein
RMSD	Root mean square deviation
SCLC	Small cell lung cancer
SER	Serine
SI	Selectivity index
SMILES	Simplified Molecular Input Line Entry System
STAT3	Signal transducer and activator of transcription 3
THR	Threonine
VAL	Valine
