# Supplementary material for: Panduratin A from Boesenbergia rotunda Effectively Inhibits EGFR/STAT3/Akt Signaling Pathways, Inducing Apoptosis in NSCLC Cells with Wild-Type and T790M Mutations in EGFR
Source: Int J Mol Sci. 2025 Mar 6;26(5):2350. doi: 10.3390/ijms26052350 (PMC11900324; doi:10.3390/ijms26052350)
Supplement: Supplementary file 1 [file ijms-26-02350-s001.zip › ijms-3473746-supplementary.pdf]

Supplementary material

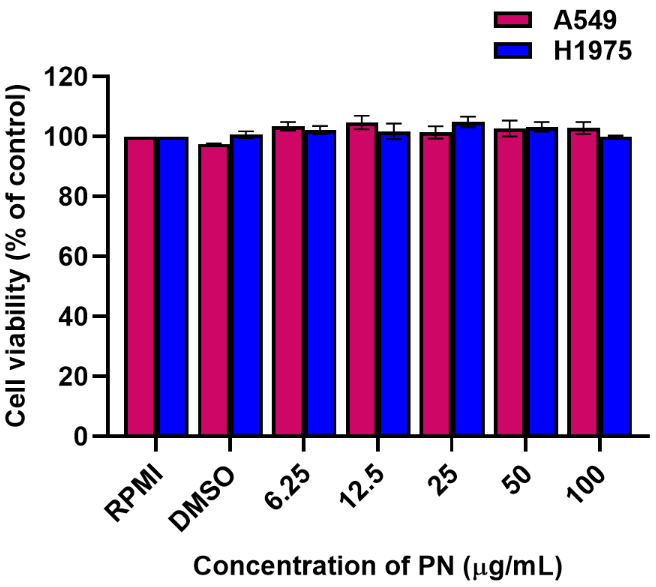

(a)

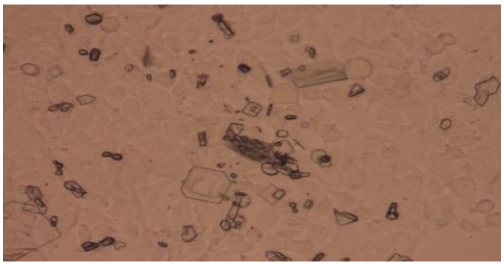

(b)

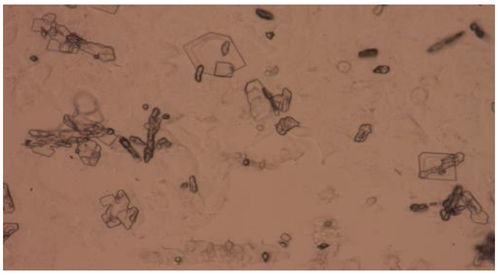

(c)

Figure S1. (a) Cell viability of A549 and H1975 cell lines after treatment with various concentrations of PN for 24 h. (b) Crystals formed in the cell culture media of A549 and (c) H1975 after treatment with 50 μg/ml of PN for 24 h. The data are expressed as the mean ± standard error of the mean (SEM) from three independent experiments.
